# Supplementary material for: Mitochondrial volume fraction and translation duration impact mitochondrial mRNA localization and protein synthesis
Source: eLife. 2020 Aug 7;9:e57814. doi: 10.7554/eLife.57814 (PMC7413667; doi:10.7554/eLife.57814)
Supplement: Supplementary file 1. [file elife-57814-supp1.docx]

**Supplementary File 1. Yeast strains and plasmids used in this study**

Strain/plasmids Genotype[plasmid](plasmid number) Source

Strains

W303-1a MATa ade2-1 can1-100 his3-11 leu2-3 trp1-1 ura3 Lab stock

TTY1160 W303-1a TIM50 ::12xMS2tag::TIM50^3’UTR^ This study

TTY584 W303-1a ATP3 ::12xMS2tag::ATP3^3’UTR^ This study

TTY439 W303-1a TOM22 ::12xMS2tag::TOM22^3’UTR^ This study

TTY1373 W303-1a ATP1 ::12xMS2tag::ATP1^3’UTR^ This study

TTY1374 W303-1a ATP2 ::12xMS2tag::ATP2^3’UTR^ This study

TTY1377 W303-1a ATP7 ::12xMS2tag::ATP7^3’UTR^ This study

Plasmids

pSH47 Gal1p-Cre Haim-Vilmovsky et al., 2009
pLOXHIS5MS2L Haim-Vilmovsky et al., 2009
pMS2CPGFP(x4) Haim-Vilmovsky et al., 2009
pvt100-dsRed Lab stock
pFA6a-link-yomCherry-SpHis5 Lee et al., 2013

pFA6a-link-yoGFP-SpHis5 Lee et al., 2013

pFA6a-link-yoGFP- CaUra3 Lee et al., 2013
pFA6a-hphMX6 Lab stock
NHB084 IRFP-Kan Gift from Nan Hao lab

TTP076 pRS406*GPD*p-Su9-mCherry This study
TTP080 pRS405*CYC1*p-MS2-4xGFP This study

TTP133 pRS403*TIM50-MS2tag* This study

TTP134 pRS403*ER-TIM50-MS2tag* This study

TTP245 pRS403*TIM50-∆MTS-MS2tag* This study
TTP135 pRS403*TOM22-MS2tag* This study

TTP136 pRS403*ER-TOM22-MS2tag* This study

TTP155 pRS403*TIM50p-TIM50mts(1-300)-TIM50cds-flagyoGFP-TIM50ter-MS2tag* This study

TTP161 pRS403*TIM50p-ATP3mts(1-300)-ATP3cds-flagyoGFP-TIM50ter-MS2tag* This study

TTP160 pRS403*TIM50p-ATP3mts(1-300)-TIM50cds-flagyoGFP-TIM50ter-MS2tag* This study

TTP162 pRS403*TIM50p-TIM50mts(1-300)-ATP3cds-flagyoGFP-TIM50ter-MS2tag* This study

TTP145 pRS403*TIM50p-TIM50mts(1-300)-TIM50cds-flagiRFP-TIM50ter-MS2tag* This study

TTP147 pRS403*TIM50p-ATP3mts(1-300)-ATP3cds-flagiRFP-TIM50ter-MS2tag* This study

TTP146 pRS403*TIM50p-ATP3mts(1-300)-TIM50cds-flagiRFP-TIM50ter-MS2tag* This study

TTP148 pRS403*TIM50p-TIM50mts(1-300)-ATP3cds-flagiRFP-TIM50ter-MS2tag* This study

TTP174 pRS403*TIM50p-TIM50mts(1-300)-TIM50cds(delpolyP7)-flagyoGFP-TIM50ter-MS2tag* This study

TTP179 pRS403*TIM50p-TIM50mts(1-300)-TIM50cds(delpolyP7)-flagiRFP-TIM50ter-MS2tag* This study

TTP158 pRS403*TIM50p-flagyoGFP-TIM50ter-MS2tag* This study

TTP119 pFA6a-link-MCP-SpHis5 This study

TTP153 pFA6a-link-MCP-CaUra3 This study

TTP167 pRS405CYC1p-MS2-2xGFP-CaaX This study
