# Supplementary material for: Mitochondrial volume fraction and translation duration impact mitochondrial mRNA localization and protein synthesis
Source: eLife. 2020 Aug 7;9:e57814. doi: 10.7554/eLife.57814 (PMC7413667; doi:10.7554/eLife.57814)
Supplement: Supplementary file 2. [file elife-57814-supp2.docx]

**Supplementary File 2. List of oligonucleotides used for plasmid construction**

Gene Name Oligonucleotides used for plasmid construction

*MS2-GFP(x4)* 5'-GACTAGTCGGATGCAAGGGTTCGAATCCCTTAGCTCTC-3'

5'-CCGCTCGAGGGCCGCAAATTAAAGCCTTCGAGCGTCC-3'

*TIM50-Tag* 5'-CCTTATTTGAAGAGGAAAAGAAAAAGAAGAAGATTGCTGAATCCAAATAAA
ACGCTGCAGGTCGACAACCC-3'

5'-ACATACACACATAGATACGTAGATACATGAGAAGAGGGTTTACATGAAAAG

CATAGGCCACTAGTGGATC-3'

*RPL2-Tag* 5'-ATAGAATGAAAGTAAAAGACAGACCAAGAGGCAAAGATGCAAGATTATAAA

ACGCTGCAGGTCGACAACCC-3'

5'-AACGAAGAAAAGGTCCCTATGTTGCTTCTTTTCTACTAAAATTAGCCTATGCA

TAGGCCACTAGTGGATC-3'

*ATP3-Tag* 5'-TTACTAATGAACTGGTTGATATTATTACTGGTGCTTCCTCTTTGGGATGAAAC

GCTGCAGGTCGACAACCC-3'

5'-TTCTACAAAAACAACGTCAAATAAAGAGGCAATGCAGGGTGATTTTTTTAGC

ATAGGCCACTAGTGGATC-3'

*TOM22-Tag* 5'-ATAACATATTGGCCCAAGGTGAAAAAGATGCTGCAGCAACAGCCAATTAAA

ACGCTGCAGGTCGACAACCC-3'

5'-ATGTATGGCTCCTTTTCTAAAACCCTCTCTTTTCTTTTACATCATTAAAAGCAT
AGGCCACTAGTGGATC-3'

*ATP1-Tag 5'-GTTGGCATCTCTAAAGAGTGCTACTGAATCATTTGTTGCCACTTTTTAAAACGCTGCAGGTC*

*GACAACCC-3'*

*5'-ATTTCTTTTTGAGACGTACCTTATATTCATTTTTATTTTTTTAGTTCACAGCATAGGCCACTAG*

*TGGATC-3'*

*ATP2-Tag 5'-TGAAGATGTTGTTGCTAAAGCTGAAAAGTTAGCCGCTGAAGCCAACTAGAACGCTGCAGGT*

*CGACAACCC-3'*

*5'-TACCTTCGGTATTTCAAATTTTGCTTCCCTTGGTTTAAGCTTTATTTCTTGCATAGGCCACTA*

*GTGGATC-3'*

*ATP7-Tag 5'-GGACGTACCTGGTTACAAGGACAGATTCGGCAATTTGAATGTGATGTAGAACGCTGCAGGT*

*CGACAACCC-3'*

*5'-TGTGAAAAAAATAATAGAATATGGTGCGTAATATATAGAGGTAAAGGGTAGCATAGGCCACT*

*AGTGGATC-3'
GPDp Sac2 F-680* 5'-TCCCCGCGGCAGTTCGAGTTTATCATTATCAATACTGCC-3'

*GPDp Hind3 R-1* 5'-CCCAAGCTTTTTGTTTGTTTATGTGTGTTTATTCGAAAC-3'

*color START Spe1 F* 5'-GACTAGTCGGTGACGGTGCTGGTTTA-3'

*color ADHt Kpn1 R* 5'-GGGGTACCTTACCCTGTTATCCCTAGCGGATCTGCCGG-3'

*C450_2C1_top* 5'-ATGGATCCAGTTGTCGTATTGGGTTTATGTTTGTCTTGTTTGTTACTACTTTCT
TTGTGGAAACAATCTTACGGTGGCGGAAAGTTA-3'

*C450_2C1_bot* 5'-TAACTTTCCGCCACCGTAAGATTGTTTCCACAAAGAAAGTAGTAACAAACAA
GACAAACATAAACCCAATACGACAACTGGATCCAT-3'

*TIM50 F-500 Spe1* 5'-CGGTGGCGGCCGCTCTAGAACTAGTACTGTGTTGGCTTTAACTCTTTAAATTC
TCC-3'

*TIM50 R++500 Xho1* 5'-AATTGGGTACCGGGCCCCCCCTCGAGATGTGACGGCAGTTCCTGACCTGATA

GTG-3'

*GA_TIM50_F129* 5'-CAATCTTACGGTGGCGGAAAGTTACAAAAAGAAACAAAAGACGACAAGCCT

AAATC-3'

*GA_TIM50_R-1* 5'-ACCCAATACGACAACTGGATCCATTGCAAGCGGGTGATTTTTGGAAGTTTATT

CTAGC-3'

*TOM22 F-500 Spe1* 5'-CGGTGGCGGCCGCTCTAGAACTAGTCAAAAAGAGCTAATCAACTCCTTGAAC

TTAG-3'

*TOM22 R++500 Xho1* 5'-AATTGGGTACCGGGCCCCCCCTCGAGGTTTACGTTTTAGATTACCAAAAAGG

AAGCATAG-3'

*GA_TOM22_F1* 5'-CAATCTTACGGTGGCGGAAAGTTAATGGTCGAATTAACTGAAATTAAAGACG

ATGTC-3'

*GA_TOM22_R-1 5'-ACCCAATACGACAACTGGATCCATTTGAATGATGCTTATTTTGGGGTATATAG*

*TTCCG-3'*

*GA_TIM50_F562 5'-GAGCCACCTTTCCCTGATTTACTATACCAAAGGCCATTAACTCTTGTTATCACA-3'*

*TIM50_R540 5'-TAGTAAATCAGGGAAAGGTGGCTCTTGGA-3'*

*yeGFP-CaaX_F 5'-GGTAAGGCTAGCGGTAAAAAGAAGAAAAAGAAGTCAAAGACAAAGTGTGTAATTATGTAAC*

*TGGTCGAGTCATGTAATTAGTTATGTC-3'*

*yeGFP-CaaX_R 5'-CTTTTTACCGCTAGCCTTACCAGCACTGCCTGCGCTATCGCTACCTTTGTACAATTCATCCA*

*TACCATGGGT-3'*
